# Nosological and Therapeutical Researches on Gangrene from Obstruction to the Circulation of the Blood

**Published:** 1842-07

**Authors:** 


### ART. VII.

*Nosologish-therapeutische Untersuchungen über die brandige Zerstörung durch Behinderung der Circulation des Blutes.* Von CARL F. F. HECKER.—*Stuttgart*, 1841. 8vo, pp. 71.

*Nosological and Therapeutical Researches on Gangrene from Obstruction to the Circulation of the Blood.* By F. F. HECKER.—*Stuttgart*, 1841.

WE have, in the work before us, a sensible and practical digest of the present state of science with respect to that form of mortification commonly designated "gangræna senilis," a form of disease which the author tells us that he has had many opportunities of observing. The present production includes the results of his observations, interwoven with a critical illustration of the opinions and views hitherto prevalent respecting this affection: and as gangrene from obstructed circulation is a subject of very considerable interest, and cases of it not very frequently met with in the ordinary walks of practice, we presume that, in furnishing a brief analysis of the contents of the present little volume, we shall perform no ungrateful task to a considerable portion of our readers.

Notwithstanding the efforts of distinguished pathologists of various nations, and the accumulated results of morbid anatomists, the most contradictory views continue to prevail upon many points of this enquiry.

The cause of this continued confusion rests in the common sources of error, an imperfect definition of terms, and the too eager spirit of generalization. One and the same pathological process has been discussed by authors under different designations; and particulars of individual cases have been prematurely considered as characteristic of the affection universally.

The term *gangræna senilis,* as commonly applied, is decidedly objectionable, because it leads to the belief that the affection occurs only in old persons, a proposition which experience by no means sanctions. A variety of other designations, such as gangrene of the wealthy, atrophy from closure of the arteries and veins, gangrene from obliteration of the arteries, cadaverization, gangrene consecutive to arteritis, atonic mortification, and so on, are all too confined, and alike inappropriate, as they imply conditions present only in individual cases. The expression "spontaneous gangrene" is faulty in the other direction, since it would include, besides the form of gangrene under discussion, many others of a totally different nature: besides, gangrenous destruction, strictly speaking, does not occur spontaneously, but as the result of some antecedent morbid process.

Our author, in his own definition, adopts the one furnished by our countryman Dr. Carswell in his work on Pathological Anatomy, and regards the phraseology "mortification from a mechanical obstacle to the circulation of the blood" as best expressing the essential condition of this morbid alteration. The obstruction in question may exist in the heart, in the great vessels, or in their minuter ramifications, and may be the result of various organic changes. According as the special lesions display the phenomena of acute or chronic disease, two kinds of this destructive process are noticed, the inflammatory and the atonic gangrene.

The immediate occasion of this morbid process is most frequently found to rest in such an organic change within the arteries, that the afflux of the vital fluid to a part is either rendered exceedingly imperfect or is entirely abolished; so that nutrition and the other vital actions become entirely suspended. The diminution of the arterial caliber occurs either in the great trunks or in the minuter ramifications, and very often in both at the same time. Such diminution may arise from the presence of coagula, of fibrine, of organized or organizable lymph, or of other extraneous products within the vessels, thereby causing complete or partial closure of their canals. Changes occurring in, between, and about the arterial coats themselves, may also lead to the same result. Now, although it cannot be denied that, in many cases, mortification follows these deviations from the normal condition of the blood-vessels, it is yet true that these do not always originate the affection in question; hence it is certain that some other condition, not as yet so well understood, must at the same time exist; because, in the first place, it is very often observed that extensive derangement, leading to complete or partial obliteration of the arterial canals, takes place without any corresponding production of gangrene, and in some instances without even a trace of disease manifesting itself during life; in the next place, the frequency of cases of gangrene stands in no definite proportion to that of such lesions of the vascular structure, the latter being very often and the former but rarely observed; and lastly, the intensity of the affection in particular cases does not seem to depend upon the extent to which the great arterial trunks have suffered disorganization. It is requisite then to look to alterations in the capillary system of vessels for the concurrent and more immediate cause of the mischief. M. Hecker controverts, and we believe successfully, the position assumed by Dupuytren, Broussais, Delpech, Bouillaud, and some other French pathologists, that inflammation of the arterial structure forms the proximate cause of the phenomena under discussion. Slow and gradually induced organic changes in the arterial and capillary vessels, as well as the occurrence of active inflammation in these structures, he regards as quite adequate to produce a gangrenous destruction of parts. Hinderance to the supply of blood is however essentially the antecedent condition, differences arising from the mode of its origin. Organic affections of the heart and great vessels, as also any interruption to the supply of nervous influence to a part, constitute circumstances favoring the development of mortification. Inflammation of the veins and absorbent vessels forms only an accidental though perilous complication, and is by no means, as contended by some, the immediate cause of the malady.

The *predisposing causes* of this affection concern the age, the sex, the situation of the parts, the mode of life, and the previous state of health. An interesting statistical table embodying some of these particulars, as they were found in 73 cases, is given by our author, the results of which are, as regards age, that, in 67 of the cases where the age had been recorded,

| | | | | | | |
|---|---|---|---|---|---|---|
| 1 | occurred between | the ages of | 1 | and | 10 | years. |
| 6 | ,, | ,, | 10 | ,, | 20 | ,, |
| 7 | ,, | ,, | 20 | ,, | 30 | ,, |
| 9 | ,, | ,, | 30 | ,, | 40 | ,, |
| 1 | ,, | ,, | 40 | ,, | 50 | ,, |
| 12 | ,, | ,, | 50 | ,, | 60 | ,, |
| 19 | ,, | ,, | 60 | ,, | 70 | ,, |
| 3 | ,, | ,, | 70 | ,, | 80 | ,, |
| 8 | ,, | ,, | 80 | ,, | 90 | ,, |
| 1 | ,, | ,, | 90 | ,, | 100 | ,, |

From these results, obtained indiscriminately from the observations of pathologists throughout Europe, the impropriety of the term "gangræna senilis" is obvious, although the greater proportion of cases unquestionably arise in advanced life.

With respect to sex, although Pott affirms that, of 20 individuals affected with the present form of gangrene, scarcely a female is to be found, and although this statement is confirmed by some others, as Jeanroy and Balling, it is yet certain that this is no universal fact, since in France it occurs almost as frequently in the one sex as in the other; and where the disparity seems to be great, as in our own country, it is probably owing to the peculiar habits of the respective sexes. At any rate, the ailment is not so infrequent in the female sex, according to our author's table, as it has been too readily assumed by some. Of 69 cases, 27 were females. This gives a proportion of males considerably less than two to one.

The parts of the body most frequently involved in this destructive process are those far removed from the centre of the circulating system, as the hands and the feet; it is but rarely that the nose, ears, lips, cheeks, &c. are implicated. As farthest from the heart, the lower extremities suffer more frequently than the upper, in the proportion of about three to one. Although, in the whole number of cases, this preponderance obtains, yet in the female sex the predisposition would seem to exist mainly in the upper extremities. Referring again to our author's table, it appears that of 17 cases where these latter were the seat of the disease, 10 were of the female sex and 7 of the male. It may not be certain that this result would receive confirmation from an extension of the numbers observed. If it should, however, it is matter for further enquiry, upon what this peculiarity may depend.

The previous habits of the patient, where they have been for a considerable period opposed to a right system of hygiène, furnish strongly predisposing circumstances; as also the long continuance of any pre-existing disease, inducing great impairment of the powers of life, either by direct injury done to the blood-vessels themselves, or by vitiation of the fluids of the body.

The *exciting causes* are not always to be detected. At times the endurance of a high degree of cold in the extremities, at others some mechanical injury done to the parts, seems to constitute the immediate occasion. But very often the attack appears to arise spontaneously, whence proceeds the designation "spontaneous gangrene" sometimes applied to this affection.

The progress of the symptoms may be traced through a succession of five stages: 1st, that of premonition; 2d, the inflammatory stage; 3d, the stage of gangrenous alteration; 4th, that of demarcation and throwing off of the mortified part; and 5th, the stage of cicatrization. Our author regards inflammatory action as preceding, in all cases, the actual occurrence of gangrenous alteration. In most cases no very striking morbid phenomena give warning of the approach of this fearful malady; and, although antecedent disease should exist, no material for a certain diagnosis in the early stage, is afforded. Anomalous gouty attacks often constitute the premonitory symptoms. The second stage begins with a slight erysipelatous reddening of the part affected which, from infiltration into the subjacent cellular tissue, yields on pressure, and it frequently exhibits a tense, marble-like aspect, whilst dark blue spots are remarked upon the affected surface. The principal vessels of the limb pulsate in many cases very strongly; whilst, in the smaller ramifications, little vital action can be noted. *Bruits* of various kinds can often be detected, with or without the stethoscope, along the course of the vessels; and these are often to be felt like firm stretched cords, painful on pressure. Other signs of inflammatory action soon show themselves, both generally and locally, varying in intensity according to the more or less acute character of the case. *Phlyctenæ* are next to be noticed upon the erysipelatous skin, and then the unequivocal display of mortification takes place, evinced by a gradual destruction of all the tissues of the part affected. Only very seldom, after the accession of the symptoms of disorganization, is anything like a reaction—a return to the normal condition of things—to be found. Cases of this kind, however, have been related by Van Swieten, Schenk, Lannelongue, and Vernhes de Rubastens.

The temperature of the mortified parts is ever remarkably low, as may be perceived by the simple touch. Dupuytren discovered by the thermometer that this was even below that of the dead body; and Chevalier has shown that the heat of the toes, when affected with gangrene, is always below that of the chamber in which the patient happens to be placed.

The gangrenous alteration may limit itself to one or several toes, may proceed along the foot to the ankle-joint, or even, in some rare cases, onwards to the hip-joint itself. Generally speaking the powers of life become entirely exhausted long before any such progress is obtained. A fatal advance of the affection is to be feared, if no cessation or limitation of the erysipelatous inflammation can be remarked. When, on the contrary, the morbid alteration seems for some time fixed to a place, with a gradual decrease of the redness from behind forward towards the toes, and when the accompanying œdema assumes a cooler character, the formation of the line of demarcation and the rejection of the mortified parts may immediately be looked for. This introduces the fourth stage.

A red streak is now manifested, which, in a very short time, circumscribes the parts in a state of incipient gangrene. In a few days, or even in a few hours, a furrow is noticed in the position of the red streak, from which there issues a laudable pus in tolerable quantity. Through this suppurative process, the separation of the dead from the living structures is brought about, occurring in the first instance with the skin and cellular tissue, whilst the fibrous, tendinous, and vascular structures, together with the nerves and the bones, resist for a considerable period the gangrenous destruction. The nervous tissue maintains its vitality the longest, on which account a high sensibility of the mortified part is often retained long after the apparent completion of the process. The final rejection of the dead tissues does not, as Balling maintains, always occur at a joint, for this will not rarely be perceived in the continuity of bone. We have ourselves observed this to take place at the junction of the middle and lower third of the tibia. Parallel cases are recorded by Chelius, Nicksius, Regnault and some others. Upon the attainment of this result, the patient's health generally improves; and it is sometimes most remarkable how vigorously the digestive process keeps up under such circumstances. When an unfavorable issue ensues, it is generally characterized by low typhoid fever.

The fifth stage, or that of cicatrization, presents no remarkable phenomenon; it pursues, with various delays and interruptions, much the same course as in cases of amputation. False anchyloses very commonly form in the joints near to the mortified parts, especially when these happen to be the elbow or knee joint.

When this malady arises in the aged, or in constitutions prematurely worn out, it occurs most usually in a chronic form. In such cases an organic change in the arterial parietes is the most ordinary concomitant. The formation of the line of demarcation, the process of rejection, that of cicatrization, and so on, are in their characters essentially the same in chronic as in acute cases. When the disease exists in children, its progress is much the same as in adults. Jäger, who has observed this form of disease in young children, is of opinion that, in some instances where infants are born with defective extremities and where clear marks of cicatrization seem discoverable at the stump, the defect is attributable to gangrenous destruction occurring to the fœtal limbs whilst in utero. The water canker of children our author excludes from the present category of disease.

The duration and progress of the affection differ so completely in different instances, each case so exhibiting its own specialities, that nothing definite upon this branch of the subject can be established. Sometimes the process of destruction advances, with unspeakable suffering, in a few days from the toes to the knee-joint, or even to that of the hip; whilst, at other times, months are required to complete the morbid alteration in one or more toes; the difference seeming to depend mainly upon variations in the exciting cause. It is certain that when arteritis, or other inflammatory ailment, occasions its development, the progress is more rapid than when it takes its origin in other organic changes gradually arising. The formation of the line of demarcation, and the rejection of the mortified part, advance with a course corresponding with that of the anterior gangrenous destruction.

*Diagnosis.* Although no difficulty exists in establishing the correct diagnosis after the appearance of the mortification itself, there is yet sometimes considerable embarrassment in determining the nature of the affection whilst in the first and second stages. The necessity of being able to form something like an accurate judgment at the onset, however, becomes the more obvious, when it is considered that otherwise the subsequent gangrene will be almost sure to be attributed to the treatment employed by the practitioner. Here our author slightly reviews the various diseases with which the first symptoms of the malady may be confounded. The most prominent of these are the various forms of erysipelas. The true erysipelas (Rosa) is commonly ushered in by symptoms of gastric fever, with a throbbing sensation of heat and tension locally, but does not give rise to the same violet or blue spots, as in the affection now under consideration. In the former case the *phlyctenæ* are not filled with a similar turbid, fetid fluid. The erysipelatous affection, moreover, yields readily in most cases to appropriate treatment. In many of these instances, however, considerable difficulty may exist; and, under such circumstances, it will be well to offer a guarded opinion until the local symptoms have sufficiently declared themselves to lead to a more certain diagnosis. In such doubtful examples, the age, the constitution, the sex, the previous habits of life, and so on, must receive a very careful attention. Some confusion may occasionally exist with respect to certain other pathological changes; but, excepting in the instances of erysipelas, the difficulty need be but slight.

The *prognosis* is at all times exceedingly doubtful, but it is not by any means so unfavorable as it is commonly assumed to be. The statistical table before referred to exhibits a proportion of recoveries to the deaths as 43 to 26, a result sufficiently favorable to be at variance with opinions current with respect to the fatality of this disease. In the establishment of a prognosis, regard must be had to all the antecedents and accompaniments; and, even when this is done with the greatest care, a *probable* judgment can alone be formed. The acute form of the disease is at all times more dangerous than the chronic, especially if it run a rapid course, and occur in a constitution previously injured, either by dissipation in early life or antecedent disease. The chronic affection is the more suspicious according as the subject of it is advanced in life, or as the vital energy is exhausted from any cause whatsoever. Death is usually preceded by symptoms of low typhoid fever, and the character of the local disease is mainly distinguished in such cases by an absence of successful attempts at the formation of the boundary line. A succession of two or three attacks is not unfrequently recovered from, but the happy issue of a *fourth* is a rare phenomenon.

*Treatment.* In the treatment of the present species of gangrene, prophylactic measures are too much neglected, though, after all, they constitute probably the most important feature. All exciting causes, so far as may be practicable, should be withdrawn, and a judiciously conducted hygiène be strenuously enforced. Specifics calculated to prevent the invasion of mortification do not exist, or at least are totally unknown; and the intention of the practitioner must be directed to the obstruction of its progress, the facilitation of the process of separation, and the mitigation of the suffering associated with the organic change. The treatment, as a whole, must aim at, first, the subduing of the phlogistic process; secondly, the favouring of the limitation of the disease, and the rejection of the dead structure; and lastly, the obtaining of a healthful cicatrization, and the removal of any induced deformity which might afterwards limit the function of the parts. The treatment will, however, vary considerably according to the difference in the immediate cause of the disease. If the patient be not far advanced in years, and if the morbid phenomena indicate the presence of acute inflammation in the affected structures, an antiphlogistic treatment will of course be appropriate. This may either be local or general according to the special circumstances of the case. When, in these instances, the use of leeches is indicated, the practitioner must be careful to apply them at some distance from the seat of inflammation along the course of the arterial vessels, as otherwise the irritation caused by the leech-bites might constitute a source of great aggravation. The employment of opiates and other anodynes, both topically and generally, is highly advantageous in almost all cases.

In the chronic form of the complaint all depressing measures must generally be avoided. The sunken powers of life have rather to be supported as much as possible by the employment of such tonic remedies as the peculiarities of the case may indicate. The employment of the preparations of bark combined with some slight acid, as the phosphoric, will usually be attended with advantage. Diffusible stimulants are rather injurious than otherwise in these cases. The local treatment is commonly best conducted by the application of aromatic fomentations and poultices, rendered slightly acid by the admixture of vinegar. Scarification and other analogous means are studiously to be avoided. Notwithstanding that Mott, Crisp, Brulatour, and some others have amputated above the diseased part with advantage, the instances in which this has occurred are, in the estimation of our author, rather to be regarded as fortunate accidents than as recommendatory of the practice; and, in this opinion, he is borne out by the opinions and the experience of most surgical authorities.

Having furnished a brief summary of the contents of the little volume before us, we must, in conclusion, express our great satisfaction with the work, taken as a whole. Almost everything that is known of this disease is here given in a concise form, together with some excellent observations proper to the author himself. We have some doubts regarding the strict pathological identity of the various cases included by M. Hecker in his category. Mortification occurring in old age, the result of what may be called a senile degeneracy in the texture of the arterial coats, we yet think, is entitled to be regarded as a form of disease sufficiently well marked to suggest the propriety of a classification apart, under the old term of "gangræna senilis." We think, also, that a greater pathological difference will be found amongst the cases included by our author than between some of them and others which he would reject as not properly embraced within his adopted definition. However, there is so much of the practical to approve in the present production that we forbear to press any theoretical criticisms which may seem to detract from our general commendation of the work.

---

